# Higher blood pressure control rate in a real life management program provided by the community health service center in China

**DOI:** 10.1186/1471-2458-14-801

**Published:** 2014-08-07

**Authors:** Xiao-Jing Chen, Xi-Lian Gao, Gui-Ying You, Jing Jiang, Xiao-Lin Sun, Xiao Li, Yu-Cheng Chen, Yu-Jia Liang, Qing Zhang, Zhi Zeng

**Affiliations:** Department of Cardiology, West China Hospital, Sichuan University, Chengdu, Sichuan China; The Yulin Community Health Service Center, Chengdu, Sichuan China

**Keywords:** Hypertension, Blood pressure, Disease management, Public health, Community

## Abstract

**Background:**

Community health service center (CHSC) in China is always regarded as a good facility of primary care, which plays an important role in chronic non-communicable disease management. This study aimed to investigate the blood pressure (BP) control rate in a real life CHSC-based management program and its determinants.

**Methods:**

The study enrolled 3191 patients (mean age of 70 ± 10 years, 43% males) in a hypertension management program provided by the Yulin CHSC (Chengdu, China), which had been running for 9 years. Uncontrolled BP was defined as the systolic BP of ≥140 mmHg and/or the diastolic BP of ≥90 mmHg, and its associated factors were analyzed by using logistic regression.

**Results:**

The duration of stay in the program was 33 ± 25 months. When compared with the BP at entry, the recent BP was significantly lowered (147 ± 17 vs. 133 ± 8 mmHg; 83 ± 11 vs. 75 ± 6 mmHg) and the BP control rate was dramatically increased (32 vs. 85%) (all p < 0.001). The age of >70 years [1.40 (odds ratio), 1.15-1.71 (95% confidence interval)], female gender (0.76, 0.63-0.93), longer stay of >33 months (0.77, 0.63-0.94), doctor in charge (0.97, 0.95-0.99), and the use of calcium channel blocker (1.35, 1.09-1.67) were significantly related to uncontrolled BP at the recent follow up (all p < 0.05).

**Conclusions:**

This CHSC-run hypertension program provides an ideal platform of multi-intervention management, which is effective in achieving higher BP control rate in community patient population. However, the BP control status could be affected by age, gender and adherence of the patients, as well as practice behavior of the doctors.

## Background

Hypertension is a major cardiovascular disease and confers the highest risk attributable to cardiovascular death [1, 2]. Low rates of awareness, treatment and control on hypertension pose a very serious public health problem worldwide, in particular in China and other developing countries [3]. Blood pressure (BP) control rate is suggested to be closely associated with provider response to uncontrolled BP and patient adherence to treatment recommendation [4–6]. Therefore, the measures to improve both aspects should be considered, such as training of care provider, communication between care provider and patient, patient education, self-management and family support [6–15]. In the last decade, China has started to integrate hypertension control into its health care reform policy that emphasizes both prevention and management of chronic non-communicable diseases. Consequently, community health service center (CHSC) as a major model of primary care in China is activated to implement comprehensive management program for hypertensive patients living in the community [16–21]. The use of community health facilities has been widespread for non-communicable diseases and reported in high-income countries but there are almost no studies from low- and lower-middle-income countries such as China [22]. Therefore, the present study aimed to evaluate the effectiveness of a CHSC-based BP management program in China and analyze the determinants of suboptimal BP control in this multi-intervention modality.

## Methods

### Hypertension disease management program

This program was established in 2004 when the Yulin CHSC (Chengdu, China) was selected as a sample center of chronic non-communicable disease management in China. In 2007, the management of hypertensive patients was included as a basic service item of every CHSC by the national laws [19, 20]. The scope and content of the study program were therefore modified according to the national regulations. Basically, the program provides 4 times of scheduled face-to-face follow-up annually and one check-up for every registered patient. However, more frequent follow-up is encouraged among the patients, e.g. once a month. Each follow-up contains multiple essential interventions: (1) BP measurement; (2) assessment of symptoms; (3) heart rate and weight measurement; (4) record of compliance on lifestyle modification and medication; and (5) prescription and patient education. This management program was approved by the local ethics committee of the Yulin CHSC and its cluster.

Of note, it is a real life disease management program, in which the patients are allowed to come in or drop out at any time and not a pre-specified antihypertensive regimen is adopted. Therefore, initial and maintenance treatment plans for the study patients are made by the doctors based on their clinical judgments and changes of medication are allowed at any time in the program. However, in case of refractory hypertension, hypertension urgencies or significant comorbidies, the patients should be referred to local hospitals of higher levels. The principles of face-to-face patient education at each follow-up are to emphasize adherence to medication and lifestyle modification, however, it is allowed to be individualized between doctor and patient. The management program also provides open classes of patient education on hypertension, usually once a month. Furthermore, a third part is invited to conduct quality control in the program, by giving phone calls to randomly selected patients for details of the services they have received. The primary BP target of the management is set to the 140/90 mmHg criteria for all participants in the program regardless of their comorbidies, which appears simple and feasible in the CHSC setting [3, 23, 24]. Furthermore, the BP control rate under this cutoff value becomes the parameter of management efficiency evaluated by the CHSC authority.

Baseline characteristics of the patients and the BP measurements during follow-up are stored in a dedicated database for the program. The diagnosis of diabetes is established according to concurrent guidelines [25, 26]. The habit of smoking or drinking is recorded according to the patient’s self-report. Smoking is graded as “never”, “light” (≤10 cigarettes/day) and “heavy” (>10 cigarettes/day), in which the latter two are labeled as “yes”. Drinking is graded as “never”, “infrequent” (≤twice/week, >1 glass of wine or equivalent for each time) and “frequent” (>twice/week), in which the latter two are labeled as “yes”.

### Patients

Potential candidates of the management program are those with essential hypertension and the age of ≥35 years, who live in the Yulin community. The hypertensive patients (treated or not treated) found at family medicine clinic at the CHSC, regular health promotion program and home visit in the residential areas were all invited. Unfortunately, some subjects did refuse to participate with unaddressed reasons but the number of patients who refused was not recorded. Those patients who agreed to participate were recruited in the program and each of them was asked to provide an informed consent. As a result, the number of patients in the program has been increasing over the years, from several hundreds to several thousands. Until the data collection, 3389 patients were enrolled in the program and the rate of drop-out during the 9 years was 3.3%. Although the reasons of drop-out were not recorded for each case, they were mainly poor compliance, move out of the community and referral to higher level hospital due to disease complexity.

### Doctors

Fourteen family medicine doctors at the Yulin CHSC have been taking care of the recruited hypertensive patients in the program. Among them, 12 (86%) doctors completed a full-time 5-year medical program with a bachelor’s degree in medical school while the other two doctors had 3 years of medicine related training with a medical diploma in technical college. All the doctors also received a part-time training in family medicine for 6 or 12 months. Standardized training was provided to the doctors on how to run the program prior to its commencement and whenever needed throughout the program. However, real practice behavior of the doctors are based on their own clinical judgments, but not pre-specified by the program.

### Data collection and analysis

This study is an analysis of the data collected prospectively in the BP management program since its commencement in 2004. The data for analysis were exported from the program database in May, 2012. The patients with the duration of stay in the program <1 month or the times of follow-up <2 were excluded. Those who remained in the program for >1 year but had no records of visit from January to May, 2012 were regarded as drop-out cases and therefore excluded. The first BP measurement was labeled as the BP at entry, while the highest office BP measured during January to May, 2012 was labeled as the recent BP. The BP measurements during follow-up were grouped by the calendar year after entry (as the 1st, 2nd, 3rd, 4th, 5th, 6th, 7th, 8th, 9th year after entry). Optimal or controlled BP was defined as the systolic BP (SBP) of <140 mmHg and the diastolic BP (DBP) of <90 mmHg, while suboptimal or uncontrolled BP as either the SBP of ≥140 mmHg or the DBP of ≥90 mmHg [3, 23, 24]. This was indeed the BP target predefined by the management program for all recruited patients. The times of visit and optimal BP control in each year were recorded for each patient, as well as the highest SBP and DBP (regarded as the SBP and DBP of the year). Subsequently, the control rates of SBP and DBP in the study population were calculated according to the SBP and DBP of the year in each patient, respectively. In addition, the demographic data, comorbidities, and BP-lowering medications at the last follow-up were collected.

### Statistics

The data were analyzed using a dedicated software (SPSS for Windows, version 17.0.0, SPSS Inc., Chicago, Illinois, USA). Continuous variables were expressed as mean ± standard deviation, while categorical data were summarized as frequencies and percentages. Paired student-*t* test or Pearson Chi-square test was used when appropriate. Multivariable logistic regression was adopted to find the associated factors of the recent BP control status. A p value of <0.05 was considered as statistically significant.

## Results

Finally, 3191 patients were included in the data analysis (Table 1). The majority of patients (2177 out of 3191 patients, 68%) had uncontrolled BP with the mean SBP of 147 ± 17 mmHg and the mean DBP of 83 ± 11 mmHg at entry.

**Figure 1:**
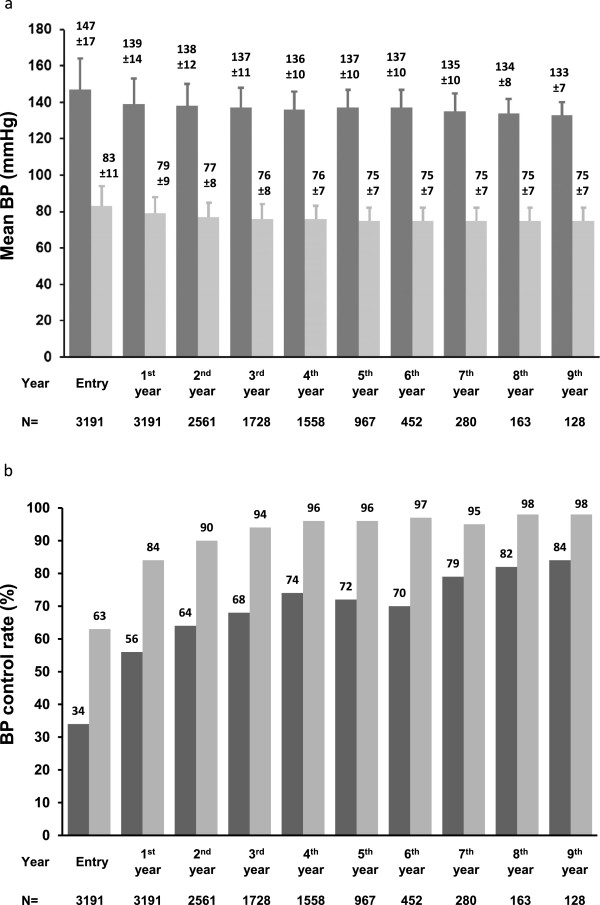
**Blood pressure control over the years in the program.** The mean blood pressure (BP) (systolic, dark grey bar; diastolic, light grey bar) **(a)** and the BP control rate (systolic, dark grey bar; diastolic, light grey bar) **(b)** of the study population over the years in the program. N, the number of patients. Data labeled as mean ± standard deviation.

The mean duration of stay in the program was 33 ± 25 (1 to 100) months for the study population. 1196 (38%) patients had stayed for less than 24 months, 1544 (48%) patients had stayed for 24–60 months, and 451 (14%) patients had stayed for more than 60 months. The visits from the 1st to 9th year were 6 ± 4, 7 ± 5, 7 ± 4, 6 ± 4, 6 ± 4, 6 ± 4, 4 ± 3, 5 ± 3 and 3 ± 1, respectively. Both the SBP and DBP during follow-up were reduced (Figure 1a) over the years after entry, and the control rates were improved (Figure 1b). The total number of visit counted 67935 in the study population with 58904 (87%) records of controlled BP.

**Table 1:** **Baseline characteristics of the patients enrolled in the program**

Patients	n = 3191
Age, years	70 ± 10
≤65 years, %	30
66-79 years, %	54
≥80 years, %	16
Gender, male vs. female, %	43 vs. 57
BMI, kg/m^2^	23 ± 3
SBP at entry, mmHg	147 ± 17
Uncontrolled SBP (≥140 mmHg) at entry, %	66
DBP at entry, mmHg	83 ± 11
Uncontrolled DBP (≥90 mmHg) at entry, %	37
Uncontrolled SBP and/or uncontrolled DBP at entry, %	68
Diabetes, %	32
Smoking, %	12
Drinking, %	14

*BMI*, body mass index; *DBP*, diastolic blood pressure; *SBP*, systolic blood pressure.

**Figure 2:**
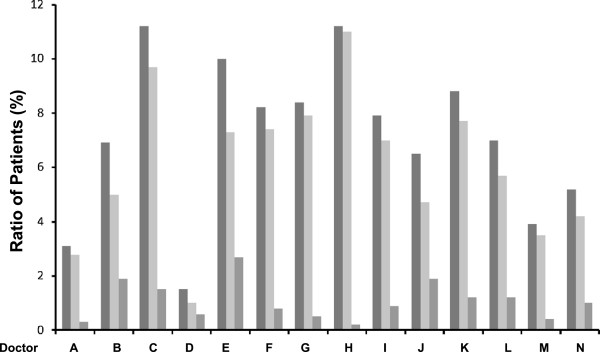
**Differences in practice of the doctors.** The ratios of the patients under care (dark grey bar, left), the patients with controlled blood pressure (BP) (light grey bar, middle) and the patients with uncontrolled BP (grey bar, right) among the 14 family medicine doctors involved (labeled as A to N) in the program.

The number of patients who were taking antihypertensive medication at the last follow-up counted 2782 (87%). 2058 (65%) patients were receiving calcium channel blocker (CCB), 841 (26%) patients were receiving angiotensin converting enzyme inhibitor or angiotensin receptor blocker, 447 (14%) patients were receiving β blocker, 121 (4%) patients were receiving diuretic and 237 (7%) patients were receiving other medicines. Among them, 823 (26%) patients were put on a combination therapy of two or more drugs. The recent SBP was 133 ± 8 mmHg with a reduction of 14 ± 17 mmHg, while the recent DBP was 75 ± 6 mmHg with a reduction of 8 ± 12 mmHg after entry (both p < 0.001). By using the recent BP, uncontrolled BP was found in 485 (15%) patients, with uncontrolled SBP in 458 (14%) patients or uncontrolled DBP in 98 (3%) patients. Only 377 out of 2177 (17%) patients with uncontrolled BP at entry remained in the uncontrolled group at the recent follow-up. On the other hand, 108 of 1014 (11%) patients who had the controlled BP at entry were found to have the SBP reading of ≥140 mmHg and/or the DBP of ≥90 mmHg during the recent follow-up (*χ*^2^ = 23.854, p < 0.001). Furthermore, differences in patient management by the 14 doctors were also found, in the number of patients under care, patients with controlled BP and patients with uncontrolled BP (Figure 2).

When compared between diabetic (n = 1030, 32%) and non-diabetic (n = 2161, 68%) patients in this study, the SBP achieved in the program was not different (133.2 ± 8.1 vs. 133.2 ± 7.7 mmHg, p = 0.894), but the DBP was lower in the former group (74.8 ± 6.4 vs. 75.6 ± 6.5 mmHg, p = 0.002). The BP control rate did not show any difference between the 2 groups according to the 140/90 mmHg criteria [3, 23, 24] of this management program (84% vs. 85%, p = 0.639). However, if the American Diabetes Association recommendation of 130/80 mmHg [27] as the optimal BP target was applied to the patients with coexisting diabetes, the BP control rate became much lower in diabetic than in non-diabetic patients (40% vs. 85%, p < 0.001).

In order to investigate the determinants of suboptimal BP control (the 140/90 mmHg criteria) at the recent follow-up (optimal BP as “0” and suboptimal BP as “1”), a multivariable logistic regression model was created including demographics, comorbidity, medication at the last visit, doctor and patient adherence to the program. As shown in Table 2, the patient age of >70 years indicated more difficult BP control, while the female gender and duration of stay in the program of >33 months were related to higher BP control rate. Not surprisingly, different effects on the BP control status were observed in the practice of individual doctors. Furthermore, the use of CCB was more involved in patients with uncontrolled BP (all p < 0.05). Interestingly, the coexistence of diabetes was not found to be associated with BP control in this model, nor the habit of smoking or drinking.

**Table 2:** **Univariable and multivariable logistic regression for uncontrolled blood pressure**

		Univariable analysis		Multivariable analysis	
Variables	Uncontrolled BP, %	Odds ratio	p value	Odds ratio	p value
Age group					
>70 years (n = 1780)	17.1	1.40 (1.15-1.71)	0.001	1.42 (1.16-1.74)	0.001
≤70 years (n = 1411)	12.8				
Gender					
Female (n = 1819)	13.7	0.76 (0.63-0.93)	0.006	0.78 (0.64-0.95)	0.013
Male (n = 1372)	17.2				
Body mass index					
>25 kg/m^2^ (n = 482)	14.9	0.98 (0.77-1.25)	0.867	1.00 (0.78-1.28)	0.978
≤25 kg/m^2^ (n = 2709)	15.2				
Duration of stay in the program					
>33 months (n = 1564)	13.5	0.77 (0.63-0.94)	0.009	0.69 (0.56-0.84)	<0.001
≤33 months (n = 1627)	16.8				
Family medicine doctor, A to N*		0.97 (0.95-0.99)	0.036	0.96 (0.94-0.99)	0.003
Diabetes, yes		1.05 (0.86-1.29)	0.639	1.00 (0.81-1.24)	1.000
Smoking, yes		1.06 (0.79-1.41)	0.716	1.27 (0.63-2.62)	0.523
Drinking, yes		1.03 (0.78-1.36)	0.838	0.77 (0.38-1.57)	0.470
Calcium channel blocker, yes		1.35 (1.09-1.67)	0.005	1.42 (1.14-1.77)	0.002
ACEI or ARB, yes		1.12 (0.90-1.39)	0.305	1.18 (0.94-1.48)	0.150
β blocker, yes		0.76 (0.56-1.02)	0.067	0.75 (0.56-1.02)	0.066
Diuretic, yes		1.25 (0.81-1.92)	0.310	1.57 (0.95-2.55)	0.069

*ACEI*, angiotensin converting enzyme inhibitor; *ARB*, angiotensin receptor blocker; *BP*, blood pressure.*The family medicine doctors involved in the program are labeled as A to N, as in Figure 2.

## Discussion

This study demonstrated that case management of hypertension by a CHSC in China helped to improve BP control among more than 3000 hypertensive patients in a local residential community. It was among the very few investigations on a real life disease management program rather than a pre-specified treatment study. During the mean stay of nearly 3 years in the program, the SBP was reduced from 147 ± 17 mmHg to 133 ± 8 mmHg and the DBP from 83 ± 11 mmHg to 75 ± 6 mmHg. The BP control rate achieved by the program was 85%, significantly higher than the 32% at entry. Shorter duration of stay in the program, older age and male gender were found to be the major determinants of suboptimal BP control.

CHSC has gradually become a major model of primary care in urban China since 1997, when the general policy framework of health care reform and specifications of health facilities started to be constructed [28, 29]. There was a fast growth in the number of CHSC during the last decade that reached 7861 in 2011 and 84% of them were run by the government [21]. More than that, the scope of services provided by CHSC to local residents has also been expanding, including health education, family planning, immunization, rehabilitation and chronic disease management. Case management of hypertension as a serious chronic illness is mandated by the “Specifications of National Basic Public Health Services” promulgated in 2007 and reinforced in 2009 [20]. This legalistic document sets out a number of specifications with regard to target population, type and frequency of service as well as evaluation of service. Therefore, it becomes a routine work of CHSC to screen, treat and follow up hypertensive residents in a dedicated management program regulated by the national laws. Thirdly, investment and subsidy in CHSC from the government have markedly increased, and more proportions of health facilities in CHSC are recognized by governmental health insurance or others [21, 28]. Therefore, we have to understand that the observed reduction in BP may be attributable to time-related changes in medical system and society system in addition to the management program *per se*.

The national health care policies consolidate the infrastructures of CHSC services to hypertensive residents, however, the success of this program could also be explained by some other factors. Like many community health service centers in China that transformed from previous local small hospitals or clinics, the Yulin CHSC has its own pharmacy, biochemistry lab, family medicine clinic, day care bed and health promotion unit. It provides an ideal platform for team based multi-intervention management that suggested to be the most effective model in BP control [6, 12, 15, 30–35]. Being a single-center study, the representativeness of this study could be limited to the area where the study was performed. However, there are large areas in Asia and Africa where similar situation exists and this study could be useful to those groups. Unlike many treatment (pharmacological or non-pharmacological) studies with predefined inclusion criteria, exclusion criteria, intervention and follow-up period, this real life hypertension management program provides an easy access to a long-term care for a general population with the disease. Therefore, the participants would be activated to adhere to the program for their own benefits. Currently, the Yulin center serves the community with 80734 registered residents and there are 44028 residents aged ≥35 years. In contrast to the high control rate achieved in the program, the enrollment percentage of applicable residents (<10%) was much lower than the prevalence of hypertension in general Chinese population [3]. Of note, the percentage of patients aged >65 years was as high as 70% that not exactly comparable to the distribution in general population. This could be attributed to the facts that most patients with serious complications and comorbidies were treated at higher level hospitals and CHSC was not yet accepted as the first choice by some patients, in particular younger generations with better medical benefits from their workplaces. However, more systematic and effective ways to screen and register hypertensive patients living in community would be urged.

The scope of services provided by CHSC to local residents includes chronic disease management and some other aspects such as health education, family planning, immunization and rehabilitation. Therefore, the doctors in the study do not contribute equally to different parts of work at the CHSC, which explains the heterogeneous distribution of the registered hypertensive patients among the doctors. Although they had similarly good education and training background, the 14 doctors could differ in their practice performance that may impact on the BP control of their own patients. Therefore, it may not be overemphasized the importance of complying with the current international and national practice guidelines among community health service providers. Unlike the generally low training level of community health service staff in China (only 28% of general physicians have Bachelor degree), the doctors involved in the study are undoubtedly able to provide better services to their patients [36]. This would definitely be helpful of obtaining patient satisfaction with the center’s services, in particular, their adherence to the management program. Interestingly, a longer duration of stay in the program was associated with a higher BP control rate. This was rarely investigated in previous community based studies, but indeed emphasized the importance of receiving persistent management among hypertensive patients [37–40]. Therefore, they should be informed, educated, encouraged and reminded of adhering to the program.

### Limitations of the study

Firstly, the recruitment of study subjects who were largely a self-selected group may limit the representativeness of the findings. However, compared with a treatment study with many inclusion and exclusion criteria, this real life management program would be more representative of a general population that indicated by the distribution of patients among different age groups and both genders. Secondly, it is useful of recording the response rate to the program and the likely reasons for not participating. The lack of data on mortality and the reasons of drop-out may not be excused by the relatively low drop-out rate either. Thirdly, the lack of control subjects in this study makes it difficult to evaluate the actual effect size of BP management program. However, the “absolute” control rate of 85% achieved in the program is still promising by comparing to low control rate in hypertensive population. Certainly, it would be better to collect more information on the change of medication, diabetic control, renal function, lipid profile, amount of alcohol intake and amount of tobacco consumption, though its feasibility would be challenged in a CHSC program.

## Conclusion

The CHSC-based multi-intervention management program for community hypertensive patients, reinforced by the national health care reform policy of China, shows its effectiveness to achieve a higher control rate in a more general patient population. The BP control status in the program is associated with adherence of the patients and practice behavior of the doctors.

## Abbreviations

CHSC: Community health service center
BP: Blood pressure
SBP: Systolic blood pressure
DBP: Diastolic blood pressure
CCB: Calcium channel blocker.
